# American Academy of Dental Science

**Published:** 1899-11

**Authors:** 

**Affiliations:** American Academy of Dental Science


### AMERICAN ACADEMY OF DENTAL SCIENCE.

THE regular monthly meeting of the American Academy of Dental Science was held at Young's Hotel, Wednesday evening, May 3, 1899, at six o'clock.

A paper was read by Edwin T. Darby, M.D., D.D.S., of Philadelphia, entitled "Dental Education a Problem."

(For Dr. Darby's paper, see page 705.)

DISCUSSION.

*President Cooke.*—I will call on Dr. Kirk to open the discussion.

*Dr. Kirk.*—I had not an idea of what Dr. Darby's line of treatment of this subject was to be, and I had hoped that I should be able to hear what Boston has to say about it before Philadelphia was laid under further contribution.

I cannot appreciate what your interest is in this matter, but if it is similar to my own, it seems to me to be the most burning question that we have before us as professional men. In the early part of the essay my heart began to run down into my shoes with the feeling that perhaps Dr. Darby proposed to maintain that we were asking too much of our dental students. It is certainly in evidence, both from what has been presented to us in the essay and also from the observed facts in the case, that dentistry has grown, and it simply means that the dental practitioner to-day must be a more highly educated man than he was years ago when dentistry was new. Some old wiseacre, I have forgotten who he was, originated the saying that "every man is the architect of his own fortune." Another has paraphrased that with the statement that "every dentist is the architect of his own professional career," and the simile is a very good one. But the question suggests itself, Are we actually making architects in the profession of dentistry? It is clear to my mind that unless a man is scientifically educated in all that pertains to his profession, and has a thorough understanding of its fundamental principles, he cannot be called an architect. He may have a relation to the profession, but it is not an architectural relation; in some cases, perhaps, the relation might be described as that of hod-carrier or stone-mason. But I might go one step further and say that we have to be more than architects, for there is a wide difference between knowing how to do a thing and being able to do it, so that we have to unite both of these elements in our professional work.

As to the scientific side of it, I question if Dr. Black was not right when he said to me only last winter that we have only barely scratched the surface of the scientific possibilities of our profession; that hitherto we have been too largely a body of empirics, and have conducted our work in much the same manner as the ordinary cook manages the culinary department, and that seems to be the mental attitude of the student until he is trained differently. I think a man who has taught students will understand my meaning, for he has no doubt observed that when he is endeavoring to elucidate principles, his class is likely to go to sleep, unless he is a gifted talker; but if, peradventure, he gives them a formula for a tooth-wash, or a method of treating pyorrhœa alveolaris, or hints on the proper selection of materials, the note-books come out at once and all that matter is carefully jotted down. They are anxious to get recipes and formulæ and short cuts to results without being particularly interested in principles. I am glad to say that that sort of an attitude does not last throughout the course, because it very soon becomes apparent to those men that they cannot successfully practise unless they know something about principles, and I believe that to be the correct method of teaching,—to equip our men with fundamental principles, so that they may be the architects of their own profesional practice.

Now, as to the question of eliminating studies from the course as now given to the dental student. I do not believe that one of these could be safely dropped from the curriculum. I believe that the curriculum should be still further broadened. I am willing to admit that the time we have allotted to us for the dental course is all too short. We need an extra year, not only to add some features to the basis of professional training, but we need it for another purpose: we need it in order to give an opportunity to a certain selected class of men who are increasing in numbers; men who will be willing to take up lines of original research. It is quite impossible for the busy practitioner to devote the time or energy necessary for this work,—and both of those elements are needed in order to accomplish much in this line,—but there is not a man within the sound of my voice who will not recognize the dire need for men who will be willing to devote some time and energy to professional original research which will accrue to the advantage of all dental practitioners. The time to get that work done is before a man becomes loaded down with the cares and obligations of practice; and I believe that if we had the fourth year added to our dental course we could induce a fairly good number of men to take up and continue the work of original research. The objection might be raised that those men would not be properly trained for such work. That may be true, but they would at least have the ability to follow out the suggestion and work under the supervision of men who are interested in such work.

It is not Utopian, in my judgment, that we should have some method of testing an applicant to find out whether he is a suitable man to enter upon the study of dentistry, of "selecting timber," as the essayist has suggested. I believe that the time will come when we can select our dental timber to that end. I believe that some satisfactory test will in time be evolved so that we may know how to properly select men who will give assurance of becoming good dentists, and who will also be capable men for carrying on the work of dental education; but until that is done, we at least have this mode of relief,—that is, it should be the duty of all the colleges during the freshman year to make it a test year, a screen, as it were, by which to sift out those who are not likely to become successful dentists and retain those who are fit to go on. If our professors and instructors are men who really have the interests of the profession at heart, they can readily pick out the men who are fit to proceed; and those men who are incompetent to learn what is required of them should be advised, as soon as that condition is known, to give up the profession of dentistry and take up something which would be more within the range of their mental attributes.

The question of technical training is one in which I have very great interest. I believe that the technical course of instruction in the laboratory has been one of the most satisfactory additions to the curriculum that we have ever had. To show you what the results of modern technical training are upon the raw student, I would like to mention a special case which came under my observation. The man I refer to matriculated at the University of Pennsylvania at the beginning of the year now closing, and as he came to the desk I saw before me a man perhaps forty years old, with a frank, open-eyed expression, and it developed that by trade he was a locomotive engineer. The qualifications that he presented as evidence of sufficient preliminary education were somewhat of a patchwork variety. He had gotten his education here and there in an irregular way, at odd times, but when put together the sum total was sufficient to meet the requirements demanded by the school for admission. The man showed the evidence of being resolute in his purpose and of being determined to accomplish what he was about to undertake, which of course was in his favor; but what struck me more particularly during the whole interview was the method by which he picked up the pen to sign the matriculation book. He handled it so clumsily that my heart failed, and in response to my question as to whether he had any mechanical training, he said he had had none whatever, except in relation to heavy machinery. However, he was so honest in his purpose that I determined to give him a trial. I called the teacher who had charge of the instruction in the technical department and said, "I want you to keep an eye on this particular man at his work. I am anxious to know what sort of progress he is going to make. He may be able to get hold of the intellectual side of what is required of a dentist, but I have my doubts about the technical part. I am afraid that he has reached the point when his muscles are set, and that he is going to fail, so I want you to pay particular attention to him and let me know how he gets along." He was kept under close observation, and for the first two or three weeks the instructor reported that it was a decided case of raw material, and that he would not have much hope were it not for one quality that the student possessed,—that of "stick-to-it-iveness." He was patient. He was determined to do his work well. He has now had four months of technical training, and to-day I consider him one of the best-trained students in the technical department. By perseverance he has overcome that clumsiness which first caught my eye, and I mention this case at length to show you how systematic training has developed what will be a good operator at the end of the course out of apparently unpromising material. I have nothing to add to this, except to emphasize again the importance of a fourth year to give us time to teach more thoroughly the principles which the dental student should know; to allow him to become more perfect in the technical part of his work, and especially for the purpose of encouraging the work of original research.

*Dr. Smith.*—I have been very much interested in the excellent paper presented by our distinguished guest, Professor Darby, but it is a most difficult matter to grasp the salient points of a paper in listening to it once, especially when a man comes to a meeting after a hard day's work, and the machinery of his mind is not up to its usual condition.

I understood our essayist to say that our profession was an honorable profession, equal to any of the other professions, and I assent that it is an honorable profession; but I do not assent that at this speaking it is equal to the position occupied by other professions, notably the professions of law, medicine, and divinity; and why? Simply and solely because it lacks the preliminary intellectual training. Dr. Darby would be content for a time with the present conditions; he seems to be content with the graduate of the high school. But other professions are not content with the high school graduate, and just so long as we content ourselves with that or with something less as a requirement for admission into our schools, just so long will we occupy a less honorable position in the estimation of the public. The better schools of medicine, law, and divinity are graduate schools, and there is no earthly reason why in a short time our dental schools should not be graduate schools, and require a degree of letters or its equivalent to enter upon the study of dentistry. I will admit that many a man who may possess his A.B. is not fitted at all for dentistry. Very likely he would not make a success in medicine or surgery. Possibly he would make a good minister. If so, that is the profession he should enter.

There is no denying that technical training is needed to give our students the knowledge to do that which will be required of them in their practice. I sometimes feel that the curriculum as it stands in our schools to-day has been brought in at the expense of technical education. Briefly I would map out this course for the candidate for the dental school: I would advise that he have a degree from a technical school, like the Institute of Technology or any of our polytechnic schools. This would give him a technical training sufficient to enable him to readily grasp the mechanical side of dentistry, and also give him an intellectual training equal to that obtained by the study for the degree of Master of Arts. The idea of a liberal education to-day is different from what it used to be, and the best educators are not agreed as to what should be required of the student.

Assuming, then, that the dental student should be a man of letters or its equivalent, what next? I cannot agree with Professor Darby's idea that he should also be a graduate of medicine. I may be a heretic among you, but I do not believe it is necessary for a man who intends to practise dentistry to hold the degree of M.D. before commencing the study of dentistry. The holders of the better dental degrees to-day are better educated in medicine than were the older graduates of medicine in their day. Now, if a man has to be a graduate of medicine before entering on a course in dentistry, it means that he must take a four years' course, granting that he goes to the better schools. Those familiar with the curriculum of the medical schools are aware of the contention that exists among the educators in those schools, and know that there is a great variety of opinion as regards medical education itself. The dental student on his way to the dental degree should have for his first year thorough courses in anatomy, physiology, chemistry, both general and physiological, histology, embryology, and bacteriology. In his second and third years his time should be given to studies bearing directly on his special work; special care and much time given to his technical training. Time and again have I seen men of good intellectual development, bachelors of arts, masters of arts, and graduates of medicine, fail of graduation in dentistry because they were unable at the close of their work in the dental school to come up to the standard in dental work; and when they find that they have been left over, they get thoroughly indignant, and I have had them say to me, "Dr. Smith, I cannot understand this. It is the first time in my life that I have ever fallen short in an examination." They fail to grasp that technical work cannot be gained so easily as book knowledge.

I assent most fully with the ideas expressed by Professor Darby, and also by Professor Kirk, that our course should be extended, for I believe we are requiring too little from our students at the present time of that which they should know; at the same time, some advantage might be gained by increasing the length of time given to some studies and decreasing the time given to others. For instance, I think that in some schools anatomy for the dental student might be cut a little; also physiology; in fact, some medical teachers think they might be cut for the medical practitioners. But before we attempt to bring about this four years' course, I want first to see the short-term schools come up to the long-term schools. The long-term schools in their three years' course give twenty-seven months' instruction, and if the short-term schools did add another year to their course, the time of instruction which they would give to their pupils would then only be equal to what the long-term schools are now giving.

So, to sum it up, what I want to impress upon you is, first, the importance of general education to lift our profession to the same recognition as the other learned professions. I admit that it is an honorable one, and I want to see every man in it willing to stand erect, with head up, and say, "I belong to the dental profession, and I am proud of the profession and its members, for I know that they are doing an immeasurable amount of good in the relief of pain and the promotion of the general health of mankind." Next, I would like to see the short-term schools come up to three years, of nine months each. I think we may rest there until we can bring about the requirement of a degree of letters, or its equivalent, for entrance to dental work. The minute you do that, gentlemen, you have dignified your profession; you have put it on a level with the other professions, because then the *personnel* of the dental student is the equal in training and culture of the *personnel* of the students in all other professions. Then, when the fourth year is added, it should be added strictly and solely as a technical course.

*Dr. Kirk.*—There was a point brought out in the last speaker's remarks regarding which, if I understood the gentleman correctly, I shall have to take issue with him.

He stated, as his experience, that men who have taken the degree in letters and who have gone thereafter to a dental college have met their first failure to pass an examination, and that the failure has always been along the lines of technical work, and he attributes this to the fact that they do not understand that technical work is harder to absorb than book knowledge. I quite agree with him as to the condition, but disagree with him as to the diagnosis.

Some years ago I made an attempt to learn to ride the bicycle. It was a hard struggle, for the bicycle seemed to have a natural antagonism towards me and refused to be conquered, and I never succeeded in riding with any feeling of security. In view of my experience, I have stood and looked with wondering amazement at the ragamuffins and urchins riding on almost any part of the wheel without apparently giving any thought to the management of it. I began to observe the thing somewhat, and I found that every man I knew who had taken up the bicycle after he was thirty years old developed that anxious expression known as the bicycle face, and was constantly on the alert for difficulties, and I think we have in this question of learning to ride the bicycle a condition quite analogous to what we have in the matter of the technical training of students. There is, during youth, a period of muscular impressionability, and unless that is taken advantage of in its plastic period, we can never secure results as satisfactory at a later period. That fact was recognized years ago by Sir John Tomes. I was astonished when I came to read the record of that man's thought on dental education. He clearly recognized years ago the fact that there was an impressionable period, when the muscles could be trained to a greater degree than if delayed until later in life; and that thought is the foundation of all technical education. It is very much easier, as you know, to move a growing thing than it is to move a thing which has reached its type limit. That principle is fully recognized and taken advantage of in orthodontia. Taking all these things into consideration, it seems to me that the reason why those men who have had sufficient intellectual training to take the degree of A.B., and yet have failed in their technical examination, was because their technical training was taken up too late in life.

I object to the idea in general that technical training should be concentrated in the fourth year. It would perhaps be all right to leave some special cases until the fourth year, but the student must have manual training long before the fourth year, or else he is a dental failure.

There is one other point regarding which there is an apparent difference, and that is the question of preliminary education. If there is any question that we want to get into right line, it is the question, What do we mean by high standing? What is the minimum that we will accept as the proper standard for entrance into dentistry? Shall we require the same standard as is required by schools of divinity? I think Dr. Darby's suggestion, that we require a certificate of graduation from a high school, or its equivalent in examination, is the proper point for the beginning of the dental education.

*Dr. Smith.*—I presume, talking at random, that I have misstated what I believe. I thought I had expressed myself clearly, yet some of you seem to have misunderstood me, particularly in regard to technical education. I believe that the preliminary training should be gotten in a technical school. I should be very much pleased if we could get our candidates for admission from such schools as the one the gentleman has mentioned,—the Institute of Technology,—but just at present I fear that is asking too much, for the graduate of that school has received just as much mental training on his way to his degree as does the student on his way to the A.B. degree in Harvard College, while at the same time, he has acquired a great deal of technical skill. But I believe that we should demand more than a high school certificate. Now, the Harvard school will not admit any one on a certificate of any high school, for two reasons,—viz., first, it does not look right; secondly, they do not accept such certificates in the better schools of law, medicine, or divinity.

*Dr. Fillebrown.*—I want to endorse emphatically the suggestion of Dr. Kirk, that we have the burning question before us. New systems of education are coming up, and by and by we shall come out into the open field, where the view and the way is clear. Out at Cambridge there are a dozen or more courses which a student may take, each one different, and each brings the same and equally honorable degree of A.B. A person to-day taking the examinations in all the courses would require at least twenty years to do it.

Harvard is also having this same upheaval in regard to medical education, and is coming nearer to the plan proposed by President Eliot. Medicine is becoming so broad that no man can compass it within a reasonable length of time. President Eliot's plan is to establish a university of medicine, which shall teach all that has to do with animal life, in health and sickness. There are certain studies which must be taught as a basis, such as anatomy, physiology, and chemistry, but when a student has learned those, then he may branch off into the special field in which he intends to practise. No man should be expected to know as much as his professor does about each study,—it is a mistake to expect it. It is the teacher's duty to impart to the student whatever it is necessary for him to know of the subject which he is teaching. If the student can get the conclusions and understand the principles on which they are based, then it is unnecessary for him to go over the whole field. Take, for instance, physiology, in which it has been stated here this evening that the student is required to make one hundred and twenty experiments. It is not reasonable that a man should go over the whole ground if twenty-five or thirty experiments will cover the part relating to his specialty. So much of each of the basal studies as will relate to his specialty should be taught to each man; then when he is given the special instruction regarding the field in which he is to practise, he will be just as well educated as the next man, and just as honorably so. It is proposed in this university of medicine to bring all the branches under one administration; then a man will go in and select his course, and others will select theirs, and yet each will have the honorable requirements for graduation, the same degree, with perhaps a certificate which shall indicate to the public what specialty that man is accomplished in. Another advantage the university system of education has in giving the same knowledge of principles to all the men is the fact that, if the student is not quite certain which branch he is best fitted for, the first two or three years of his education will not be lost.

Technical education, I believe, should commence in the preliminary course. At the same time that we are working for the A.B., we should also endeavor to get a certain degree of technical training. Even earlier than this a person can demonstrate that he is or is not capable of becoming a good dentist.

*Dr. Hamilton.*—There is one point that I think is too good ground for us to neglect to sow a little seed. I feel from my own experience, and from that of others, that there is a possibility of a dentist suffering from nervous strain from defective vision, and I feel that a student should be examined by a competent oculist. If there is a defect, it might not be enough to unfit a man for another occupation, but still would unfit him for dentistry, and he should be advised of the fact.

*Dr. Werner.*—I hope that if we reorganize our methods of teaching, the technical training will come in among the first year studies. I think anybody who has had anything to do with students, or who reflects on his own career, will see the importance of that. Physiology interests all educators. Neither does a purely medical education suit us best. We need a mixture of a mechanical, surgical, and medical curriculum at our dental schools.

*Dr. Eames.*—The question of physical disability, raised by Dr. Hamilton, is an interesting one, to which I have given some attention. If these physical defects could be determined before the student enters college, it is so much the better for him and for the school. I remember the case of a man applying for admission who had lost two fingers. He was not debarred from entrance, because it was possible that he might be able, notwithstanding this loss, to do as well as some other man with the full complement of fingers. During the first year's training at the school these infirmities and disabilities should be taken into consideration, and whatever else would disqualify a man should be found out during this time.

As to the matter of what we shall require of our candidates for admission, when we state in our catalogue that they must give evidence of the equivalent of a high school education, we have in mind a certain standard of high school. Now, the standard should be high and broad; for it is necessary that there should be a certain discipline and training of the mind. I care not where the individual gets it, or in what particular study, be it in Latin, mathematics, or physics, but he needs a certain amount of mental training to enable him to grasp the pathological questions with which we have to deal. It seems to me that too little time is given to finding out what the result of our teaching has been, and to watching the development of students under our care; I believe, also, that quizzing and clinical work should occupy a larger part of the teaching hours.

*Dr. Andrews.*—In the matter of education, I think we are drifting to just what Dr. Fillebrown has told us,—an idea I have advocated for many years,—that is, to the development of a medical university which shall teach all branches, medicine, dentistry, and other specialties, and shall require the same entrance examination from all those who wish to enter, and shall give the same basis of instruction to all; but giving, in addition, special courses, so that the student may fit himself for any branch in which he may wish to practise. It is simply applying the university idea to the study of medicine and its specialties. A man in Harvard University is obliged to take eighteen courses to get through. Another man may take entirely different courses, and yet get the same degree. It should be thus in a medical university. It seems to me that if a man goes through Harvard University with the idea of taking up a profession afterwards, he can find courses there which will educate the fingers as well as the brain, and which will be of advantage to him in whatever profession he desires to enter.

I thoroughly believe in this idea of technical education for the man who wishes to become a dentist, and that the student should be tested as to his capability in this direction. It should be found out in the first year whether he is capable of performing his duties as a dentist, and if he does not show this element of technical ability, he should be dismissed and not allowed to continue.

*Dr. Wilson.*—A good many years ago, when Dr. Harwood was president of this Society, he made the statement that dentists ought to serve an apprenticeship as sculptor, and also as jeweller, before beginning to practise their profession.

I also remember that Dr. White, in an address before a graduating class, insisted that no man should enter the medical school unless he had graduated from some college, and there are a great many who believe that all dentists should be graduates of medical colleges.

Now, if these statements are true, and a man should try to comply with all these conditions, he would be at least thirty years old or more before he would be able to start out in life and build up a practice. As Professor Darby says, the element of time is of considerable importance, and perhaps the question of how to get a dental education in a reasonable time will be solved if the system of education referred to by Dr. Fillebrown as being advocated by President Eliot is brought into existence. I firmly believe, however, that the ultimate result will be, that a special degree will be given, showing that a man is fitted for the special calling in which he is engaged. I think Dr. Smith has been misunderstood in his advocacy of an additional year in the dental school. I do not believe, myself, and do not think that he intends to convey the idea, that the technical training in the regular course should be any less that it is at the present time.

*Dr. Smith.*—Neither do I. I have tried twice to make my position clear on that point.

*Dr. Wilson.*—I am constantly surprised by the small amount of practical knowledge possessed by graduating students. I am one of those who think the medical degree is not essential. It is a good thing to have a good, broad education, and the more a man knows the better; but in all educational matters the element of time must be considered. It seems to me that the best way to advance our profession is to raise the standard of our dental schools.

*Dr. Fillebrown.*—What we want this evening is to make this discussion practical. I would like to ask one question of any of the gentlemen here advocating more thorough instruction in techniques, and, particularly, of Dr. Kirk. The most of our schools commence about the first of October. Now, is a month's strict attention to technical work sufficient to test the student's technical ability? If so, why not take the month of September, and have our students come and give a little attention to that?

*Dr. Kirk.*—I should not regard a month as sufficient if you want to thoroughly test a man's capability to do technical work. Of course, you may get a superficial idea by observing how he does things. In the case that I spoke of, the manner in which that student picked up a pen made me question whether he had technical ability, and it was really some weeks before I was satisfied upon this point.

*Dr. Fillebrown.*—How many weeks did it take to satisfy you that he had the element you were looking for?

*Dr. Kirk.*—Not less than six weeks.

*Dr. Fillebrown.*—He was attending to other forms of work at the same time, was he not?

*Dr. Kirk.*—Yes: the usual studies of the school.

*Dr. Fillebrown.*—If he was not so occupied, it would shorten the time to find out what you wanted to three or four weeks. I am proposing to take him before the other studies begin.

*Dr. Werner.*—In some European schools, metallurgy, the physical working of metals, and dental technique are taught in the first year of the course.

*Dr. Kirk.*—A great many American schools teach them in the first year.

I would like to add one more word. I feel that I am taking up a great deal of your time, but the subject is so intensely interesting to me, I trust you will pardon me for so doing. The idea referred to by Dr. Fillebrown as having been suggested by President Eliot,—that of founding a university in which everything relating to animal life is taught, and the giving of a degree to those taking prescribed courses,—I think is entirely correct, and if the same principle is applied in the preliminary education before we come to the dental course, I should consider the plan a very desirable one.

Now, let us see where we stand to-day as between dentistry and medicine. Dr. Fillebrown has said that there are a few studies which form the basis of the education of all those who study any branch of medicine; and he named anatomy, physiology, chemistry, as some of them. But why stop there? Why not get at exactly what they are? It seems to me simply a question of defining what those fundamental studies shall be. In dentistry to-day we already have those studies as a part of our course. Anatomy, physiology, chemistry, pathology, histology, osteology, embryology, and therapeutics are taught on precisely the same basis as they are taught in the medical schools. We have that; it is already accomplished. The question which we really have before us is, to define the difference between the complete medical curriculum to-day and the proposed medical curriculum which shall be sufficient for the needs of the specialist. Many of the studies, we know, are organically the same, and what is now needed is some systematic organization of the several curricula leading to each of the medical specialties, including dentistry; for I hold that even now, the dental curriculum of our best schools is equivalent to that required of other specialists in the healing art.

*Dr. Darby.*—Just a word in regard to the preliminary education. I had in mind the high school of the better quality when

I said that should be one of the first conditions required of the student before matriculation. I did not mean by that, that the time would not come when we should demand something more; but I did not want to be in the position of the man who in his boyhood days had a friend who afterwards became President of the United States. He thought it would be a good plan to renew the old acquaintance, and in the course of his examination with the President he asked if he would grant him a favor. The President replied, it would depend upon what it was. He said that he would like to be ambassador to the Court of St. James, but was told that the position was already filled. He then asked to be minister to France, but the President replied that they had a good man there. He next inquired about the position of minister to Italy, consul-general to Egypt, and various other positions, and in each case received the answer that the places were already filled with good men. But he did not propose to go away without getting some evidence of the President's friendship, so he finally said, "Perhaps you have an old suit of clothes that you will give me." So we must not ask too much in the beginning, and then come down. Some of our colleges are receiving men without any fitness whatever for professional life; men who cannot write a single sentence correctly; men who cannot spell the word California correctly. We are compelled to go slowly in raising our standard of admission. No one would be more pleased than I to see our schools requiring the degree of A.B. or A.M. as an essential for entrance; but we cannot get it this year, and probably shall not next year; but if we can within the next few years arrive at the position where we can demand that our candidates for admission shall come to us with a well-rounded education, such as would be furnished by one of your Boston high schools, I should think that we were making progress in the right direction.

In the matter of eliminating from the dental curriculum all or part of certain studies, I am afraid that I was misunderstood. What I intended to say was, that it is the duty of the schools to curtail somewhat their curriculum, and I believe it could be done without detriment to the student. There are some studies which might be shortened, and the time given to studies which are of more importance to the dental student. If we have but three years in which to educate our dental students, would it not be better to devote at least half of that time to the studies which will fit him for the practice of dentistry? The dental student to-day spends a year and a half of his three years in acquiring a knowledge of branches which are certainly desirable for the professional man to know; but it does not afford him sufficient time to gain that knowledge which he should have of operative and mechanical dentistry. It is a nice thing to know anatomy from head to foot, and physiology is also an interesting and valuable study, but a man cannot become a good physiologist, a good anatomist, a good microscopist, and everything else in three years.

Now, in regard to the fourth year, in which I advocated that practical work should be taught. I did not say that practical work was not to be taught until the fourth year. A man's technical training should begin as early as possible in the first year, and his development in that direction should be regarded as of great importance. My idea was that the fourth year should be added, so as to give the student a year for practice in operative and mechanical work.

I wish to thank you, gentlemen, for the interest which you have shown in my paper.

*Dr. Fillebrown.*—I want to move a vote of thanks to Dr. Darby and Dr. Kirk for making this meeting so interesting for us; and that we request a copy of the paper for publication.

Unanimously voted.

*Dr. Eames.*—I wish to say that Dr. J. D. Thomas, of Philadelphia, was prevented from being present to-night by reason of illness.

HARRY E. CUTTER, D.D.S.,
*Editor American Academy of Dental Science.*

